# Text Messaging Intervention for Young Smokers Experiencing Homelessness: Lessons Learned From a Randomized Controlled Trial

**DOI:** 10.2196/23989

**Published:** 2021-04-01

**Authors:** Sebastian Linnemayr, Rushil Zutshi, William Shadel, Eric Pedersen, Maria DeYoreo, Joan Tucker

**Affiliations:** 1 RAND Corporation Santa Monica, CA United States; 2 RAND Corporation Pittsburgh, PA United States; 3 University of Southern California Los Angeles, CA United States

**Keywords:** smokers, quitting, text messaging, homeless, young adults, cessation resources, peer support, smoking rate, smartphone

## Abstract

**Background:**

Smoking rates are significantly higher among young people experiencing homelessness than in the general population. Despite a willingness to quit, homeless youth have little success in doing so on their own, and existing cessation resources tailored to this population are lacking. Homeless youth generally enjoy the camaraderie and peer support that group-based programs offer, but continuous in-person support during a quit attempt can be prohibitively expensive.

**Objective:**

This study aimed to assess the feasibility and acceptability of an automated text messaging intervention (TMI) as an adjunct to group-based cessation counseling and provision of nicotine patches to help homeless youth quit smoking. This paper outlines the lessons learned from the implementation of the TMI intervention.

**Methods:**

Homeless youth smokers aged 18 to 25 years who were interested in quitting (n=77) were recruited from drop-in centers serving homeless youth in the Los Angeles area. In this pilot randomized controlled trial, all participants received a group-based cessation counseling session and nicotine patches, with 52% (40/77) randomly assigned to receive 6 weeks of text messages to provide additional support for their quit attempt. Participants received text messages on their own phone rather than receiving a study-issued phone for the TMI. We analyzed baseline and follow-up survey data as well as back-end data from the messaging platform to gauge the acceptability and feasibility of the TMI among the 40 participants who received it.

**Results:**

Participants had widespread (smart)phone ownership—16.4% (36/219) were ineligible for study participation because they did not have a phone that could receive text messages. Participants experienced interruptions in their phone use (eg, 44% [16/36] changed phone numbers during the follow-up period) but reported being able to receive the majority of messages. These survey results were corroborated by back-end data (from the program used to administer the TMI) showing a message delivery rate of about 95%. Participant feedback points to the importance of carefully crafting text messages, which led to high (typically above 70%) approval of most text messaging components of the intervention. Qualitative feedback indicated that participants enjoyed the group counseling session that preceded the TMI and suggested including more such group elements into the intervention.

**Conclusions:**

The TMI was well accepted and feasible to support smoking cessation among homeless youth. Given high rates of smartphone ownership, the next generation of phone-based smoking cessation interventions for this population should consider using approaches beyond text messages and focus on finding ways to develop effective approaches to include group interaction using remote implementation. Given overall resource constraints and in particular the exigencies of the currently ongoing COVID-19 epidemic, phone-based interventions are a promising approach to support homeless youth, a population urgently in need of effective smoking cessation interventions.

**Trial Registration:**

ClinicalTrials.gov NCT03874585; https://clinicaltrials.gov/ct2/show/NCT03874585

**International Registered Report Identifier (IRRID):**

RR2-10.1186/s13722-020-00187-6

## Introduction

National data indicate that 19% of people aged 18 to 25 years in the United States are current (past 30 day) cigarette smokers [1]. However, rates of smoking among young people experiencing homelessness are significantly higher, with several studies indicating that up to 70% of the population are current smokers [2-4]. They also spend a higher fraction (about 30%) [5] of their monthly income on cigarettes, compared with about 20% spent by homeless adult smokers [6]. In addition, most homeless youth who smoke report engaging in one or more high-risk smoking practices that heighten their exposure to toxins such as smoking shared cigarettes (96%), smoking discarded butts (71%) and filters (46%), and blocking filter vents (39%) [7]. Despite the high prevalence of smoking and high-risk smoking behaviors negatively impacting the health of homeless youth who smoke, evidence-based smoking cessation programs specifically designed for this population are lacking.

This paper is based on our previous research that focused specifically on young homeless smokers and supported the need for cessation programs adapted to this population. We found high willingness to quit smoking among this population but little success in doing so on their own or when using existing available cessation resources [5,8-10]. In a sample of nearly 300 young homeless smokers, almost half (43%) were motivated to quit in the next 30 days and, of those, 76% were interested in using a nicotine replacement product (eg, nicotine patches) and/or smoking cessation counseling to help them quit. The participants reported attempting to quit an average of nearly 10 times in the past year, and two-thirds of them had quit for at least 24 hours before relapsing. Most participants (79%) who had tried to quit smoking did so on their own (ie, without counseling or medication), reflecting the lack of readily available smoking cessation services for this population. As many of these young people are not connected to the formal health care sector, we identified drop-in centers as a low barrier, “come as you are” point of service entry for young homeless smokers. These centers represent an ideal setting to reach young homeless smokers who may not seek services elsewhere, and we therefore recruited the sample for this study in this setting.

We also found that homeless youth who are interested in quitting enjoy the camaraderie and peer support that group-based programs offer [8]. Therefore, we decided to use a group format to deliver a brief in-person smoking cessation counseling session at the beginning of the intervention as described below. While there is no clear consensus on the cost effectiveness of mobile health (mHealth) behavioral interventions [11,12], in this case, continued in-person contact during a quit attempt is prohibitively time- and cost-intensive, and instead support must be provided in a way that is low cost and low burden for both service providers and their clients. A text messaging-based intervention (TMI) for cessation support circumvents some of these barriers and offers a tailored approach that can be accessed anywhere and anytime. Given widespread cell phone ownership among homeless youth, phone-based support holds great potential for behavior change [13]. Although TMIs for behavior change have not been evaluated in this population, a recent study using text messaging for daily data collection among young homeless smokers found it to be both acceptable and feasible [14]. Systematic reviews of text messaging services for adolescents have shown the general acceptability and feasibility as well as improvements in preventive behaviors and adherence to medication in the case of chronic health conditions [15,16]. Furthermore, nearly all of the homeless youth in our previous study expressed interest in using their phone to receive ongoing text messages to help them quit [10]. Phone-based smoking cessation interventions have been found to be feasible and effective in other populations, as a recent meta-analysis of 22 studies found that smokers who received a TMI were more likely to abstain from smoking relative to controls across a number of outcomes, including 7-day point prevalence (odds ratio [OR] 1.38, 95% CI 1.22-1.55) and continuous abstinence (OR 1.63, 95% CI 1.19-2.24) [17]. TMIs hold great potential (even more so in the age of COVID-19) for offering ongoing support for behavior change as an adjunct to face-to-face services.

In this paper, we report on our experiences implementing the first TMI targeted at smoking cessation among homeless youth as a part of a pilot evaluation that found promising results of the TMI on smoking reduction. We lay out the lessons learned in designing appropriate and effective text messages and implementing the technical aspects of the intervention and report on the feedback from the participants regarding different aspects of the intervention. We conclude with a discussion of what worked well and what could benefit from improvement with the hope that our lessons can be of value to other, much-needed TMIs for this particularly vulnerable population.

## Methods

### Study Design and Participants

**Figure 1:**
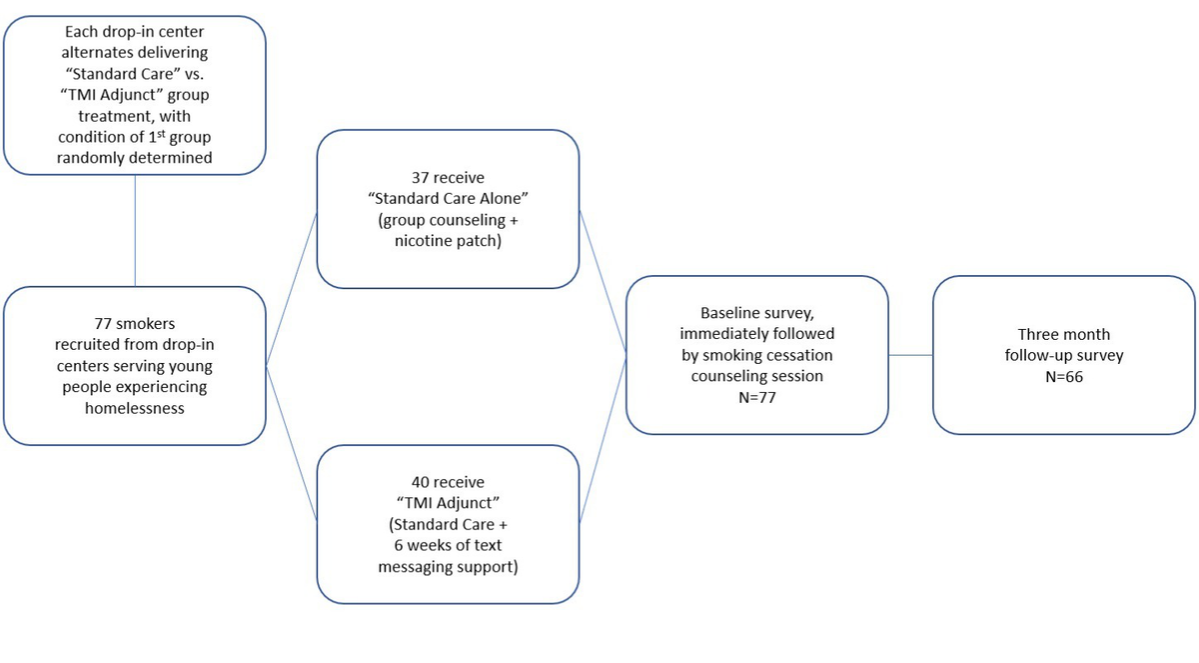
Study flow.

The findings reported here are from a pilot study of 77 current smokers aged 18 to 25 years who desired to quit and were recruited from 3 drop-in centers serving homeless youth in the Los Angeles area. The unit of analysis was the individual, but individuals were assigned to groups (standard care alone vs TMI adjunct) based on the drop-in center where they were seeking services during recruitment hours. The intervention was carried out in a cluster cross-over randomized controlled design such that each drop-in center alternated between serving as an intervention or control site by phase across the field period. After formative work with 26 participants [18], we settled on a study design that provided a brief group counseling session based on the 5 As (ask, advise, assess, assist, and arrange) [19] and nicotine patches to all participants, and those in the TMI condition were then sent automated text messages. The study flow is shown in Figure 1, and a detailed description of the intervention design has been published elsewhere [20]; here we focus on aspects related to the text messaging component. Enrollment and intervention delivery occurred between May 30, 2019, and February 25, 2020, with follow-up data collection occurring approximately 90 days later. The sample was 81% male (62/77), 71% heterosexual (55/77), and 13% non-Hispanic White (10/77). Mean age was 22.61 (SD 1.84) years. All study materials and procedures were approved by the study’s institutional review board, and a certificate of confidentiality was obtained from the National Institutes of Health. The study was registered at ClinicalTrials.gov [NCT03874585].

### Description of TMI

The project team generated 174 text messages to send to all TMI participants (see previous work [20] for example texts by the 5 main foci of the TMI program as well as TMI time point [ie, pre–quit day texts, quit day texts, early post-quit texts, later post-quit texts]). Text message content was informed by our prior work with homeless smokers to identify key factors associated with motivation to quit, consultation with the text messaging literature, and review of text messages included in other public domain smoking cessation programs (eg, Text2Quit [21] and SmokefreeTXT [22]). The majority of texts address 1 of the 5 main foci of the intervention that are based on factors identified in our prior work associated with motivation to quit among young homeless smokers [5]: strategies for getting support for quitting (“Check in with your friends and let them know how you’re doing with staying smoke-free. If you need support from them, ask for it!”); calculations for the amount of money saved by quitting (“Quitting smoking can save you some big $$! If you’re curious about how much you can save by quitting, check out this link: [link to external website]”); presentation of health and social benefits of quitting (“Sniping discarded butts might be free tobacco, but it can also make you sick from germs on the ground or from the person who smoked it first. Not worth it.”); strategies for dealing with cravings and negative moods (“Don’t lose the progress you’ve made. Ride through cravings by chewing gum, walking it off, or taking 10 deep breaths. And text CRAVE for more support anytime”); and tips for staying motivated (“Say out loud ‘I’m a nonsmoker.’ It seems cheesy, but it can remind you of all the changes you’ve made and help you stay strong through the cravings”). Given the provision of nicotine patches in this study, some texts included reminders to use the nicotine patches. Other text messages included periodic check-ins to see if they were still reading the texts (“We just want to know that you got this text. Please text back: YES”) and occasional fun content (eg, encouraging messages, emojis, or funny memes). Similar to other text messaging programs (eg, SmokefreeTXT), participants could text CRAVE, MOOD, or SLIP at any time for additional support in dealing with cravings, negative mood, or a smoking lapse, respectively; an additional 72 text messages (24 for each text type) were developed for these purposes.

It was important to ensure that the content of the text messages reflected the unique circumstances of young people experiencing homelessness. In addition, to make messages more effective we used recent insights from behavioral economics in the design of the messages [23,24], such as using gain/loss framing (“Increase your chances of success by starting to use the nicotine patches tomorrow morning”), employing social norms (“You’re not alone in wanting to be smoke-free. Most people your age don’t smoke...and most people that do smoke want to quit.”), appealing to participants’ self-identity (“Look in the mirror and tell yourself: I am a nonsmoker! Thinking of yourself as a ‘nonsmoker’ instead of an ‘ex-smoker’ can make staying smoke-free easier.”), and providing progress information to keep the salience of the quitting behavior high (“You’ve worked really hard for almost 2 weeks to get where you are now. Don’t lose this energy!”). The program was named CRUSH IT! in this spirit as well.

Given that TMIs have not been previously implemented for behavior change among young homeless smokers, there is little guidance from the existing literature regarding the frequency and timing of text messages for this population. Therefore, we conducted several focus groups with a total of 18 homeless smokers and elicited usability testing feedback with a separate sample of 10 homeless smokers recruited from the drop-in centers [18]. During these groups we also reviewed the content of texts with participants and made sure that receiving texts would be feasible for this population to inform decisions about the optimal content (eg, what types of messages would likely be most effective in dealing with cravings) and wording (eg, how to word the text message so that it motivates homeless youth to stay quit). During conceptualization of the TMI, we decided to use the online text messaging platform Telerivet to deliver the intervention. Telerivet is a free online web service that one of the authors used in previous studies [25-27]. Telerivet also offers a paid version with additional features that we made use of in our intervention. For instance, the paid version allows users to create multiple (up to 20) polls. These polls are useful for asking questions to the participants and setting up automated responses while also logging the participants’ responses. We had polls set up to occasionally check in with the participants to see if they had smoked that day and to respond with words of appreciation or encouragement based on their responses. Early on, we also used these polls to log their reason for wanting to quit smoking and sent it to them later on during the intervention to remind them of what they had said. The paid version of the platform also allows for a larger degree of automation and backend data to be collected. Since the TMI had a different start date for each intervention group, reprogramming the platform with all the messages every time would have been prohibitively labor intensive. Instead, we used Telerivet features to completely automate this process using triaged scheduling relative to the start date of each group. Telerivet also allows for a great degree of customizability to create functionalities beyond the preprogrammed options. For example, we programmed a feature allowing us to create keywords that users could send in order to get automatic supportive texts if they were tempted to smoke due to a craving or negative mood or if they had slipped and smoked a cigarette. We were also able to automate the process for allowing participants to delay the start of the intervention by a few days or stop it altogether.

### Surveys

The results in the following section come from two surveys. The first was a baseline survey that was administered when participants were first recruited for the TMI before the counseling session. This survey asked respondents for information such as demographics, phone use, smoking behavior at baseline, and other substance use. Three months after the baseline survey (and hence about 6 weeks after the end of the intervention phase), all participants received a follow-up survey that asked for information similar to that at baseline; those in the TMI condition were asked additional questions on their experience with the TMI. The baseline survey was administered in a group setting immediately prior to the group counseling session. We used self-administered paper-pencil forms, which was the most feasible option in our field setting. The follow-up surveys were administered either in person or via phone interview. Survey response forms were then scanned and checked for accuracy. While staff were available to assist participants in completing the surveys, no one required such assistance. Participants received $20 for the baseline survey and $40 for the follow-up survey. The baseline survey had 77 participants while the follow-up survey had 66 participants due to attrition.

## Results

### Lesson 1. Young Homeless Smokers Had Widespread (Smart)phone Ownership

In Table 1, we present the descriptive statistics regarding phone ownership and other phone-related parameters of participants in the standard and TMI conditions. Randomization appeared successful as there were no statistically significant differences between the two groups; although given the pilot nature of this study, we were not powered to find statistically significant differences. A key eligibility criterion was having a phone with them at the recruitment visit that could receive text messages. Confirming our assertion that we expected widespread phone ownership, of the youth who were asked about having a phone, only 16.4% (36/219) reported that they did not have one with them that could receive text messages. In addition, 3 who had a phone reported that they did not want to receive text messages (for a total of 39/219 (17.8%). Smartphone ownership was high—84% (31/37) in the standard condition and 73% (29/40) in the TMI condition owned smartphones. A majority of participants—62% (23/37) in the standard condition and 78% (31/40) in the TMI condition—had unlimited minutes. More importantly for our TMI, a majority also had unlimited texts—68% (25/37) in the standard condition and 80% (32/40) in the TMI condition. About half of the participants—40% (15/37) in the standard condition and 55% (22/40) in the TMI condition—had unlimited data. Many had obtained their phone through a benefits program such as the Lifeline program, which gives low-income Americans free cell phones, voice minutes, and texting—32% (13/37) in the standard condition and 52% (21/40) in the TMI condition.

**Table 1:** Participant cellphone characteristics at baseline.

Variables	Standard condition (answered yes; n=37), n (%)	TMI^a^ condition (answered yes; n=40), n (%)
It is my own phone	32 (86)	34 (85)
It is a phone owned by a friend, partner, or relative	5 (13)	6 (15)
It is a phone obtained through a benefits program	13 (32)	21 (52)
It is a smartphone	31 (84)	29 (73)
I have unlimited minutes on this phone	23 (62)	31 (78)
I have unlimited texts on this phone	25 (68)	32 (80)
I have unlimited data on this phone	15 (40)	22 (55)
I can view webpages on this phone because I have a data plan	27 (73)	27 (67)
I can only view webpages on this phone when I’m connected to the Wi-Fi	15 (40)	20 (50)

^a^TMI: text messaging–based intervention.

### Lesson 2. Young Homeless Smokers Experienced Interruptions in Their Phone Use But Were Able to Receive the Majority of Messages

**Figure 2:**
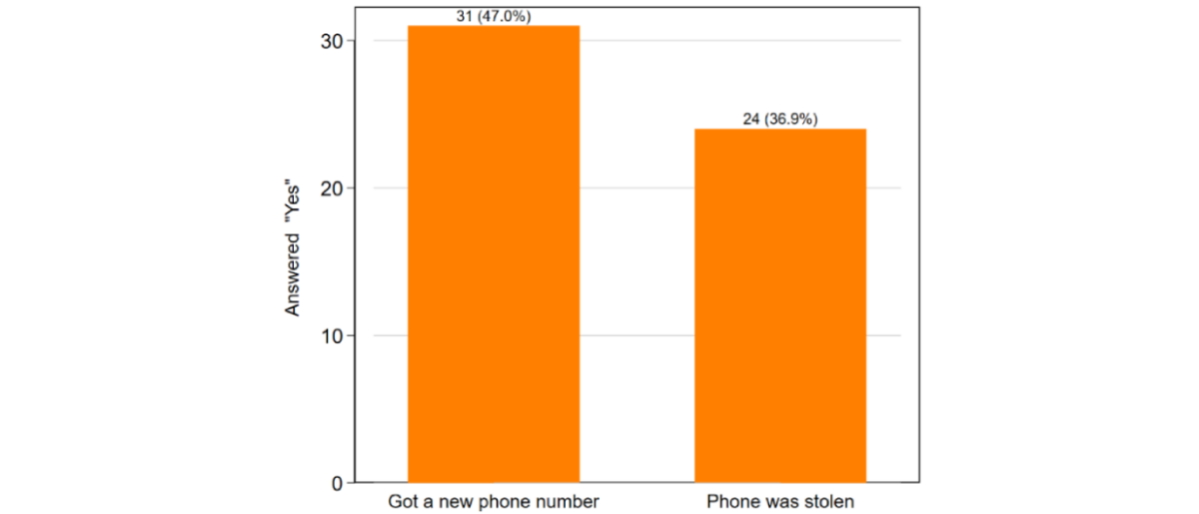
Cell phone and number retention at follow-up (n=66).

As can be seen in Figure 2, there were phone-related challenges that could influence the effectiveness of a TMI smoking cessation program. Almost half (31/66, 47%) of the sample changed phone numbers in the approximately 3 months between recruitment and the follow-up survey (15/30 [50%] in the standard condition and 16/36 [44%] in the TMI condition). Some (24/66, 37%) participants reported having their phone stolen during this period (11/30 [37%] in the standard condition and 13/36 [36%] in the TMI condition). However, based on feedback from the interviewers, a majority of participants who reported getting a new number did so between the end of the 6-week TMI period and the follow-up survey in month 3 (ie, outside of the intervention period). In addition, for participants in the TMI condition, a majority (26/36, 72%) had trouble charging their phones at least sometimes, and 33% (12/36) had no reception at some point during that 3-month period.

Despite the frequent occurrence of phone loss and switching of numbers, 81% (29/36) of TMI participants reported not having problems receiving the texts. Further, 72% (26/36) had no trouble accessing the hyperlinks provided in some of the text messages, allowing them to access supplemental information (see Table 2). Moreover, backend Telerivet data indicated a 95% message sending success rate (ie, about 95% of messages were successfully delivered to the participants on average). The reason for nondelivery of messages was either because participants’ phones were switched off for an extended period or potentially due to service issues with the carrier. A total of 55% (20/36) of TMI participants responded to at least one prompt over the 6-week period, and 11% (4/36) texted one of the unprompted keywords such as MOOD or CRAVE. Three participants proactively reached out to us to report having gotten a new phone number (participants were told at enrollment that they would receive a $5 incentive for providing updated contact information); others who got new numbers were successfully contacted using social media or other means for purposes of follow-up survey data collection. Consequently, the sample sizes between Table 1 and Figure 2 differ since follow-up data was collected for 30 standard condition and 36 TMI condition participants.

**Table 2:** Intervention delivery metrics.

Metric	Never, n (%)	Sometimes, n (%)	Often, n (%)	Always, n (%)
Trouble keeping phone battery charged	10 (28)	17 (47)	6 (17)	3 (8)
Ran out of messages and couldn’t get messages	29 (80)	3 (8)	2 (6)	2 (6)
Trouble accessing hyperlinks on phone	26 (72)	4 (11)	2 (6)	4 (11)
Did not have cellphone reception	24 (66)	10 (28)	1 (3)	1 (3)

### Lesson 3. Carefully Crafting Text Messages Led to High Approval of Most Text Messaging Components Among the Participants

Table 3 presents the opinions of participants about different TMI components. Most participants somewhat or strongly agreed that they would recommend the program to a friend interested in quitting (30/36, 83%); liked being able to text CRAVE, MOOD, or SLIP at any time to get additional support (26/36, 72%); and found that the messages helped when they were experiencing a craving (25/36, 69%). Taken together, these findings indicate participants felt that the intervention provided appropriate support for alleviating or navigating the temptation to smoke. Nearly three-quarters (26/36, 72%) of participants found the text messages helpful and said the hyperlinks provided them with useful information. Further, it appears that customizing the intervention to cater to this population was also well received—66% (24/36) reported liking the tone of the message, 78% (28/36) liked that the messages were personalized (such as by including names or referring to previous responses), and 75% (27/36) liked the use of memes and emojis in the messages. A significant proportion of participants (14/36, 44%) reported receiving the messages late because their phone was switched off or had bad reception. Further, we had open-ended questions to allow for more detailed feedback on what they liked most and least about CRUSH IT! Participants mentioned appreciating the attention to detail, the resources provided in the text messages, and the positive tone of the messages. In terms of areas for improvement, the most consistent feedback was wanting more of a group component to the ongoing support, which was mentioned by several participants. In addition, 2 participants remarked on the timing of the messages, one noted the lack of a real person for support and questions, and one thought that too many messages were sent.

**Table 3:** Intervention acceptability metrics.

Metric			Strongly disagree, n (%)		Somewhat disagree, n (%)		Somewhat agree, n (%)		Strongly agree, n (%)
**The text messages:**									
	helped when I was experiencing a craving	4 (11)		6 (17)		17 (49)		8 (23)	
	helped when I was having trouble staying quit	3 (9)		9 (27)		14 (41)		8 (23)	
	helped keep me motivated to quit	3 (9)		6 (17)		11 (31)		15 (43)	
	provided me information that I could use	6 (17)		3 (9)		4 (11)		22 (63)	
**I liked:**									
	being able to text a keyword for extra support	5 (14)		4 (11)		7 (20)		19 (53)	
	the memes and emojis that were sent	4 (11)		4 (11)		10 (29)		17 (49)	
	that the text messages were personalized	4 (12)		2 (6)		11 (32)		17 (50)	
	the tone of the text messages	6 (18)		4 (12)		12 (35)		12 (35)	
	when the messages asked me to respond	6 (18)		5 (14)		14 (41)		9 (27)	
I received text messages late because my phone wasn’t charged, or I had bad reception			14 (41)		5 (14)		9 (27)		5 (14)
Hyperlinks to websites provided information I could use			5 (14)		3 (9)		10 (29)		16 (47)
I would recommend the CRUSH IT! program to a friend who is trying to quit smoking			4 (12)		1 (3)		7 (19)		23 (64)

## Discussion

### Principal Findings

In this paper, we discussed the development of a smoking cessation TMI for young homeless smokers and reported lessons learned regarding intervention components such as development of the messages, (smart)phone ownership, technical challenges with the intervention, and feasibility (such as whether messages were received) and acceptability (ie, feedback about what intervention aspects the participants liked).

Regarding message development, we found that conducting focus groups with young homeless smokers and pilot testing with participants before rolling out the intervention were crucial steps to successfully tailoring the content, tone, and frequency of messages to the target population. Participants preferred messages that provided information specific to their current living situation, were light in tone rather than being preachy, and contained fun elements such as emojis and memes. We then programmed the messages developed with the participants’ input using a web-based text messaging platform that allowed for automatized sending of messages and categorization of participant responses for subsequent sending of appropriate follow-up messages, which minimized human resources required for implementation and guaranteed that messages were sent out with a high success rate.

As would be expected based on other research showing widespread cell phone ownership among individuals experiencing homelessness [28-30], we found that we had to exclude only a small percentage of young people experiencing homelessness from participating due to not having a phone that could receive text messages; indeed, most participants had smartphones, unlimited text and call plans, and a significant portion even had unlimited data plans. Taken together, these results suggest that phone and even smartphone-based interventions are feasible for this highly mobile population. Reflecting the challenges of cell phone ownership among individuals experiencing homelessness reported previously [31], during the intervention we realized that a large fraction of participants either had their phone stolen or switched numbers and most had trouble keeping their phone consistently charged. However, based on participant feedback they still received a majority of text messages, speaking to their resilience in the face of technical difficulties but also to the relatively short intervention duration of 6 weeks during which most participants may not have yet experienced these problems (the data were collected several weeks after the intervention ended and may have picked up difficulties arising after the intervention period).

Last, similar to prior research finding high acceptability among young people experiencing homelessness for using text messaging for daily data collection [14], we found high acceptability of our text message support for quitting smoking, with a majority of participants indicating that they would recommend the TMI to a friend trying to quit. Participants found the messages to contain information useful to them and they liked the light tone we tried to convey and the fun elements such as emojis and memes, demonstrating that the formative work and piloting of the intervention we performed before rolling it out to all participants were important to hit the right content and tone. Given increasing evidence of technology fatigue and the failure of many individuals to either take up or keep engaged with mHealth interventions [32], this is an important lesson for future such interventions. Overall, our results show that text message–based interventions can be a valuable approach to support smoking cessation efforts of homeless youth who are highly mobile and difficult (and costly) to reach with traditional means, and that the specific needs and preferences of this population need to be taken into account in the design and implementation of such an intervention.

### Strengths and Limitations

Our study has significant strengths such as being the first TMI for smoking cessation support for homeless youth and implementation at 3 sites in the Los Angeles area, which allowed for testing in different contexts. However, it also had limitations. For example, results may not generalize to homeless youth in other geographic areas or to those younger than age 18 years. Due to the study being a pilot, we were intentionally not powered to evaluate statistical differences between the standard and TMI conditions. Future studies should test the promising results of this pilot in a fully powered trial ideally in several different locations to test for generalizability of our results. In addition, it is a limitation that we did not collect qualitative data (eg, debriefing interviews) with the homeless youth who used the TMI at the end of the study, which might have identified additional strengths and weaknesses of this approach that could inform future research efforts in this area. Clearly, our study contributes to a growing evidence base that TMIs for smoking cessation can be effectively implemented even for highly transient and resource-constrained populations such as homeless youth, but that adaptation to their specific needs (including conducting appropriate formative work) is needed to render the TMI acceptable.

In terms of future directions, one specific recommendation we got from participant feedback is that they really enjoyed the group cessation counseling session prior to receiving the TMI and wanted more group elements incorporated into the intervention. Going forward, future smoking cessation interventions for this population should consider using approaches making full use of smartphone capabilities, including virtual approaches to leverage group interaction not requiring in-person meetings such as using social media chatrooms. Given their low costs and low requirements for human resources (particularly given the currently ongoing COVID-19 epidemic), (smart)phone-based interventions are a promising approach to support homeless youth, a population urgently in need of effective smoking cessation interventions.

### Conclusions

In conclusion, we find that most young homeless smokers have cell phones that allow mHealth interventions, with many being in possession of smartphones that typically have unlimited minutes and texts. Given that almost half also have unlimited data plans, it seems that in the near future internet-based interventions requiring smartphones will also be feasible in this population. In line with widespread concern in the literature, we find that homeless youth reported frequent occurrence of phone loss and switching of numbers. However, despite these difficulties the majority of participants reported not having problems receiving the study texts, and engagement with the different intervention components was generally high. We hope that the lessons derived from this pilot intervention serve as useful inputs for future mHealth studies for this population in need of smoking cessation interventions.

## Abbreviations

mHealth: mobile health
OR: odds ratio
TMI: text messaging–based interaction

## References

[1] (2017). Key substance use and mental health indicators in the United States: results from the 2017 National Survey on Drug Use and Health.

[2] Wenzel SL, Tucker JS, Golinelli D, Green HD, Zhou A (2010). Personal network correlates of alcohol, cigarette, and marijuana use among homeless youth. Drug Alcohol Depend.

[3] Baer JS, Ginzler JA, Peterson PL (2003). DSM-IV alcohol and substance abuse and dependence in homeless youth. J Stud Alcohol.

[4] Bousman CA, Blumberg EJ, Shillington AM, Hovell MF, Ji M, Lehman S, Clapp J (2005). Predictors of substance use among homeless youth in San Diego. Addict Behav.

[5] Tucker JS, Shadel WG, Golinelli D, Ewing B, Mullins L (2015). Motivation to quit and interest in cessation treatment among homeless youth smokers. Nicotine Tob Res.

[6] Arnsten JH, Reid K, Bierer M, Rigotti N (2004). Smoking behavior and interest in quitting among homeless smokers. Addict Behav.

[7] Tucker JS, Shadel WG, Golinelli D, Mullins L, Ewing B (2015). Sniping and other high-risk smoking practices among homeless youth. Drug Alcohol Depend.

[8] Mullins L, O’Hanlon CE, Shadel WG, Tucker JS (2017). A qualitative study of smoking cessation experiences and perceptions among homeless young adults. J Soc Distress Homeless.

[9] Shadel WG, Tucker JS, Mullins L, Staplefoote L (2014). Providing smoking cessation programs to homeless youth: the perspective of service providers. J Subst Abuse Treat.

[10] Tucker JS, Shadel WG, Golinelli D, Ewing B, Mullins L, Staplefoote BL (2015). Reducing cigarette smoking among unaccompanied homeless youth. Rand Corporation.

[11] Badawy SM, Kuhns LM (2016). Economic evaluation of text-messaging and smartphone-based interventions to improve medication adherence in adolescents with chronic health conditions: a systematic review. JMIR Mhealth Uhealth.

[12] Iribarren SJ, Cato K, Falzon L, Stone PW (2017). What is the economic evidence for mHealth? A systematic review of economic evaluations of mHealth solutions. PLoS One.

[13] Rice E, Lee A, Taitt S (2011). Cell phone use among homeless youth: potential for new health interventions and research. J Urban Health.

[14] Tyler KA, Schmitz RM (2017). Using cell phones for data collection: benefits, outcomes, and intervention possibilities with homeless youth. Child Youth Serv Rev.

[15] Badawy SM, Barrera L, Sinno MG, Kaviany S, O'Dwyer LC, Kuhns LM (2017). Text messaging and mobile phone apps as interventions to improve adherence in adolescents with chronic health conditions: a systematic review. JMIR Mhealth Uhealth.

[16] Badawy SM, Kuhns LM (2017). Texting and mobile phone app interventions for improving adherence to preventive behavior in adolescents: a systematic review. JMIR Mhealth Uhealth.

[17] Scott-Sheldon LAJ, Lantini R, Jennings EG, Thind H, Rosen RK, Salmoirago-Blotcher E, Bock BC (2016). Text messaging-based interventions for smoking cessation: a systematic review and meta-analysis. JMIR Mhealth Uhealth.

[18] Tucker JS, Pedersen ER, Linnemayr S, Shadel WG, DeYoreo M, Zutshi R (2020). A text message intervention for quitting cigarette smoking among young adults experiencing homelessness: study protocol for a pilot randomized controlled trial. Addict Sci Clin Pract.

[19] Shadel WG, Niaura R, Abrams DB, Niaura R, Brown R, Emmons K, Goldstein MG (2003). Brief behavioral interventions. The Tobacco Dependence Treatment Handbook: A Guide to Best Practices.

[20] Tucker JS, Pedersen ER, Linnemayr S, Shadel WG, DeYoreo M, Zutshi R (2020). A text message intervention for quitting cigarette smoking among young adults experiencing homelessness: study protocol for a pilot randomized controlled trial. Addict Sci Clin Pract.

[21] Abroms LC, Boal AL, Simmens SJ, Mendel JA, Windsor RA (2014). A randomized trial of Text2Quit: a text messaging program for smoking cessation. Am J Prev Med.

[22] SmokefreeTXT.

[23] Garcia-Retamero R, Cokely ET (2011). Effective communication of risks to young adults: using message framing and visual aids to increase condom use and STD screening. J Exp Psychol Appl.

[24] Linnemayr S, O'Hanlon C, Uscher-Pines L, Van Abel K, Nelson C (2016). Using insights from behavioral economics to strengthen disaster preparedness and response. Disaster Med Public Health Prep.

[25] MacCarthy S, Mendoza-Graf A, Huang H, Mukasa B, Linnemayr S (2019). Supporting Adolescents to Adhere (SATA): lessons learned from an intervention to achieve medication adherence targets among youth living with HIV in Uganda. Child Youth Serv Rev.

[26] Linnemayr S, Huang H, Luoto J, Kambugu A, Thirumurthy H, Haberer JE, Wagner G, Mukasa B (2017). Text messaging for improving antiretroviral therapy adherence: no effects after 1 year in a randomized controlled trial among adolescents and young adults. Am J Public Health.

[27] Rana Y, Haberer J, Huang H, Kambugu A, Mukasa B, Thirumurthy H, Wabukala P, Wagner GJ, Linnemayr S (2015). Short message service (SMS)-based intervention to improve treatment adherence among HIV-positive youth in Uganda: focus group findings. PLoS One.

[28] Jennings L, Lee N, Shore D, Strohminger N, Allison B, Conserve DF, Cheskin LJ (2016). U.S. minority homeless youth's access to and use of mobile phones: implications for mhealth intervention design. J Health Commun.

[29] Rhoades H, Wenzel S, Rice E, Winetrobe H, Henwood B (2017). No digital divide? Technology use among homeless adults. J Soc Distress Homeless.

[30] Post LA, Vaca FE, Doran KM, Luco C, Naftilan M, Dziura J, Brandt C, Bernstein S, Jagminas L, D'Onofrio G (2013). New media use by patients who are homeless: the potential of mHealth to build connectivity. J Med Internet Res.

[31] Raven MC, Kaplan LM, Rosenberg M, Tieu L, Guzman D, Kushel M (2018). Mobile phone, computer, and internet use among older homeless adults: results from the HOPE HOME cohort study. JMIR Mhealth Uhealth.

[32] Szinay D, Jones A, Chadborn T, Brown J, Naughton F (2020). Influences on the uptake of and engagement with health and well-being smartphone apps: systematic review. J Med Internet Res.

